# Consumers’ Preferences towards Bread Characteristics Based on Food-Related Lifestyles: Insights from Slovenia

**DOI:** 10.3390/foods12203766

**Published:** 2023-10-13

**Authors:** Anita Kušar, Igor Pravst, Urška Pivk Kupirovič, Klaus G. Grunert, Ivan Kreft, Hristo Hristov

**Affiliations:** 1 Nutrition Institute, Koprska ulica 98, SI-1000 Ljubljana, Slovenia; igor.pravst@nutris.org (I.P.); pivkurska@gmail.com (U.P.K.); ivan.kreft@nutris.org (I.K.); hristo.hristov@nutris.org (H.H.); 2 Biotechnical Faculty, University of Ljubljana, Jamnikarjeva ulica 101, SI-1000 Ljubljana, Slovenia; 3 VIST–Faculty of Applied Sciences, Gerbičeva Cesta 51A, SI-1000 Ljubljana, Slovenia; 4 MAPP Centre, Aarhus University, Fuglesangsalle 4 Allé 10, 8210 Aarhus V, Denmark; klg@mgmt.au.dk; 5 School of Marketing and Communication, University of Vaasa, Wolffintie 34, 65200 Vaasa, Finland

**Keywords:** bread, functional ingredients, nutrition claims, choice-based conjoint, food-related lifestyles, latent class analysis, segmentation

## Abstract

Consumers’ recognition and understanding of food characteristics can have an important role when making purchase decisions. The current study analysed consumer preferences for bread, an important food in the diets of Central European countries. The study included a conjoint experiment on a representative sample of 547 adult consumers in Slovenia. The following bread attributes: functional ingredients (chia seeds, linseed, quinoa, and Tartary buckwheat); nutritional claims (low salt, high fibre, and high protein); and other claims (organic, free from additives, flour from Slovenia, and wholegrain) were studied. The results showed the strongest relative importance for functional ingredients (a mean relative importance of 83.9%). In addition, a deeper insight into consumer preference was investigated by a recently developed modular instrument for food-related lifestyles. Latent class cluster analysis (LCA) enabled the identification of four consumer segments (uninvolved, conservative, health-conscious, and moderate) with different preferences toward selected functional ingredients, nutrition, and other claims. The results provide insights that allow for a better understanding of consumer preferences for functional ingredients and claims, and new perspectives for bread marketing to different consumer segments based on food-related lifestyles. Identifying the drivers that affect bread purchasing and consumption can support reformulation activities and product promotion in the direction of reinforcing healthier food choices.

## 1. Introduction

Unbalanced diets (i.e., those rich in salt, sugar, and fat and low in dietary fibre), together with low physical activity, have resulted in a growing incidence of non-communicable diseases worldwide [1]. Some consumer groups are becoming more interested in healthy lifestyle and food choices, but there are many challenges to achieving lifestyle changes in the general population [2]. Consumers’ recognition of the nutritional characteristics of foods could encourage them to make nutritious and healthy food choices [3,4,5]. The European Commission (EC) strives to enable consumers to identify and choose appropriate foods, and to make choices that suit their individual dietary needs [6]. While harmonised European Union (EU) legislation has ensured that consumers are informed, in ways that are not misleading, about the composition and health functions of foods [6,7], labelling of some other food properties (e.g., their sustainability, etc.) is not yet sufficiently regulated.

It should be also mentioned that consumers’ use of food labelling information is often limited. A European study showed that less than a third of consumers pay attention to nutritional information [8]. On the other hand, they like the idea of having simplified key product information on the front of the package; some studies have also indicated the importance of the use of nutritional claims (i.e., low salt, high fibre, high protein, etc.) and claims related to animal welfare, sustainability, organic farming methods, plant-based ingredients, and social responsibility (i.e., free from additives, organic, local origin, clean label, etc.) to achieve an improvement in consumer awareness and acceptance of food products [9,10,11]. It is important that such claims are not misleading, because consumers often rely on such messages [12,13].

Bread represents an important dietary component in Europe and worldwide [14]. It is a key source of complex carbohydrates and proteins, B-group vitamins, minerals, and dietary fibre—particularly in the case of wholegrain bread. However, in Europe, the term “bread” refers to a broad range of products. The composition of bread can differ to a great extent in the number of specific constituents (additives, whole grains, seeds, and pseudocereals) and nutritional composition, i.e., the amount of salt, protein, and dietary fibre [2,15,16]. Most consumers prefer to eat bread made from highly refined wheat, mainly because of the more attractive taste and textural properties in comparison to products made from whole grains [17]. Improving bread composition in terms of functional ingredients, protein, fibre, and salt content could create additional value for consumers if such properties are perceived as a benefit, and could provide marketing opportunities for food manufacturers in a competitive retail setting [2,15,18]. European legislation enables the communication of such properties to consumers in the form of nutrition and health claims [6,7].

Consumers are becoming more focused on foods with nutritional and other benefits [19]. Consequently, the trends in bread production indicate the addition of whole grains and some traditional pseudocereals to such products. In comparison to cereals, pseudocereals are non-grasses, but are used in a similar way. They are rich in essential nutrients and bioactive compounds. Adding pseudocereal grains and seeds to bread therefore offers nutritional and health benefits [20].

To explore the preferences of consumers in Slovenia for different pseudocereal grains and seeds, the study design considered four typical pseudocereals: quinoa (*Chenopodium quinoa*), Tartary buckwheat (*Fagopyrum tartaricum*), linseeds (*Linum usitatissimum*), and chia seeds (*Salvia hispanica*). The flour of the grain Tartary buckwheat has become more widely used in foods recently, and it has a much higher content of the antioxidant rutin than common buckwheat [21]. The amino acid and mineral composition of quinoa seeds revealed their high potential as a valuable ingredient in the preparation of nutritious food [22]. In comparison to white flour, quinoa seeds have higher contents of most essential amino acids, especially lysine. On the other hand, chia seeds are high in vitamin E, carotenoids, and dietary fibre, and are also an excellent source of omega-3 fatty acids [23]. Flaxseed (linseed) is a well-established functional food ingredient, mainly because of its high content of dietary fibre and α-linolenic acid [24].

Excessive dietary salt (sodium) intake is recognised as one of the key nutrition-related global public health problems [25,26]. High levels of salt in the diet is linked to elevated blood pressure which, in turn, can lead to stroke and coronary heart disease. The World Health Organisation (WHO) has identified the bakery sector as an important contributor to the salt intake of the European population [27]. This has also been highlighted in Slovenia, where high consumption and high salt content make bread a key dietary contributor to sodium intake [16]. Although a high consumption of bread can result in higher salt intakes, bread can also be a source of beneficial constituents, such as protein and dietary fibre [2,28]. These nutritional properties are typically communicated to consumers using regulated nutritional claims (low salt, high fibre, or high protein). These constituents also affect bread’s sensory characteristics and consumers’ acceptance [29], which need to be considered in reformulation activities [18]. 

Other properties can also play a notable role in consumer purchasing behaviour. For example, some consumers seek wholegrain bread because it contains a higher content of dietary fibre, vitamins, and minerals [26]. On the other hand, some food ingredients can negatively affect purchasing behaviours. For example, many consumers perceive food additives such as chemical dough conditioners as unacceptable, even if these are used to help increase dough and bread quality during the baking process, and to improve food safety [30]. We should also mention those properties which are not directly related to (nutritional) composition, for example, the use of organic, eco-friendly, and local ingredients, and ingredients claiming environmental protection and the support of local food production and the community [31,32,33,34].

It is well-established that consumers’ ability to understand food claims depends on many different factors, such as sociodemographic characteristics, nutritional knowledge, familiarity with the food, and the label format and articulation [8]. There is limited knowledge of the extent of the nutritional, production, and ingredient-specific aspects of consumer preferences related to bread. Gaining insight into consumer preferences is valuable in assessing how different attributes and their interweaving are important when purchasing bread. High heterogeneity in consumers’ preferences for bread [35] also needs to be considered. A modular food-related lifestyle instrument (MFRL) [36] has been shown to be useful in exploring different market segments and consumers’ food preferences [37,38,39,40]. 

In line with the aforementioned challenges, this study has two primary objectives: (1) to explore consumer preferences for functional ingredients, nutrition, and other claims for bread; and (2) to investigate consumer heterogeneity based on food-related lifestyles, and to provide further insights into the different segments of consumers and their preferences for different bread characteristics. Following validation of the new food-related lifestyle instrument [36] a segmentation analysis will be conducted and the identified segments will be compared with those previously reported. We hypothesized that certain consumer segments exist with specific interest in bread products that include pseudocereals with functional characteristics that are perceived as beneficial to health. Apart from this, the results may affect product management strategies for nutritious food components and the technological and sustainable aspects.

## 2. Materials and Methods

### 2.1. Survey Design and Measures

The quantitative survey was structured in six sections: (1) a choice-based conjoint study; (2) cereal intake and health perception; (3) awareness of pseudocereal; (4) buckwheat intake, and the incentives and barriers of product consumption; (5) the food-related lifestyle instrument; and (6) sociodemographic characteristics, anthropometrics, and health status related questions. 

The choice-based conjoint study was designed to investigate the respondents’ preferences for different functional ingredients, the nutrition, and other claims used for bread. Cereal intake and health perception were investigated using closed questions asking the respondents about their frequency of intake and health perception. Awareness of the selected pseudocereals was explored using a closed question with two options: “I am aware” and “I am not aware.” Additionally, two structured questions explored the participants’ buckwheat intake, and the incentives and barriers of the consumption of different buckwheat products. To investigate the participants’ food-related lifestyles, the modular food-related lifestyle instrument (MFRL) [36,41] was used (Supplementary Tables S1 and S2). This instrument has undergone cross-cultural validation [41]. Additionally, the previous version of this instrument has been extensively employed in studies related to food consumer segmentation [42]. The newly developed version of the instrument comprises three primary modules and supplementary sub-modules. In our study, we incorporated all three primary modules, which encompass aspects of involvement, tradition versus innovation, and responsibility. Furthermore, we integrated four additional sub-modules, two focusing on planning and shopping (specifically, the use of technology for shopping and accessing product information) and two concentrating on product quality considerations (namely, product origin and aspects related to healthy eating).

All the MFRL items were measured on a seven-point Likert scale from 1 (totally disagree) to 7 (totally agree). The respondents’ sociodemographic characteristics were investigated by asking about their place of residence, sex, age, household composition and income, education, and employment. Additionally, the respondents’ anthropometric and health-related information was obtained by self-reported body weight and height, health-related conditions, and self-perceived health status.

### 2.2. Design of the Choice-Based Conjoint Experiment 

The selection of attributes and levels for the choice-based conjoint study was based on a literature review of studies investigating preferences for grains and different types of breads, as well as the use of nutrition and other claims for bread [35,43]. An exploratory study was conducted using recruitment from social media (Facebook), with the purpose of determining a final set of attributes and levels for use in the choice-based conjoint study and testing the questions for correct transcription and understanding. The results of the exploratory study showed a strong preference for and awareness of the use of linseed (96.1%), chia (91.7%), and quinoa (87.8%), and more limited awareness of Tartary buckwheat (46.1%) in bread production. In this regard, the creation of the final choice-based conjoint design entailed three bread attributes: wheat bread with none or with the aforementioned pseudocereals, three nutritional claims, and four other claims, as shown in Table 1.

These conjoint choice sets were constructed using the function dcreate [44] in STATA (Version 17.0) statistical language (StataCorp LLC, College Station, TX, USA), where random selection is modified with a Fedorov algorithm to improve the D-efficiency of the experimental design [45,46,47]. The full factorial design produced 60 profiles. By using an orthogonal fractional factorial design allowing the estimation of main effects only, the number of product cards was reduced to 40. The choice experiment questionnaire asked respondents to indicate their intention to purchase one out of four presented products; this task was repeated 10 times. 

### 2.3. Sample Selection

The recruitment was done by an online panel of a market research agency in September 2019, which provided the survey results without disclosing any personal identification parameters. We used quota sampling to ensure that the structure of the study sample was comparable to the Slovenian population for age, sex, and geographical cohesion region. Participation in the survey was voluntary, and the respondents were able to exit the survey at any time without facing negative consequences. In total, 551 respondents aged 18–65 years took part in the survey. It should be noted that population in Slovenia is about 2 mio, with 1.35 mio citizens aged 18–65 years, and that the selected sample size is comparable with other national consumer studies of similar population size [48]. The margin of error ranged between 4 and 6% respecting gender and cohesion region affiliation, respectively. The data of 547 respondents who completed the survey were used in the data analysis; 4 respondents from the initial sample were removed from the analysis due to inconsistency in the reporting of their ages. To ensure the participation of bread consumers, we used a filter question asking about the frequency of their consumption of bread at the beginning of the questionnaire. Seven participants who selected ‘no consumption of bread’ were excluded from the conjoint study and did not take part in the other cross analysis.

### 2.4. Statistical Analysis

Confirmatory factor analysis was conducted to determine whether the measures of the predefined dimensions of the food-related lifestyle instrument were consistent with the reported understandings and specifications. For this purpose, we used the statistical packages condisc [49] and averc [50], developed under STATA statistical software. The convergent validity of the measurement model was assessed by the Average Variance Extracted (AVE) and Composite Reliability (CR). AVE values above 0.7 were considered very good, whereas a level of 0.5 was acceptable [51]. CR values above 0.7 were considered acceptable. Discriminant validity was assessed by comparing the amount of the variance captured by the construct and the shared variance with other constructs. For this purpose, we tested under the criteria that the levels of the AVE for each construct were greater than the squared correlation involving the constructs. Additionally, the heterotrait-monotrait ratio of the correlations (HTMT) was determined to measure the average correlations of the indicators across the constructs. The acceptable level of discriminant validity was set at <0.90, as suggested by [52]. Estimation of individual part-worth utilities for the choice-based conjoint analysis was conducted using the ChoiceModelR package [53] in RStudio (Version 1.1.456) using the Hierarchical Bayes Estimation approach. A latent class cluster analysis (LCA) with age and sex as covariates was used to identify groups of individuals based on the three core dimensions of food-related lifestyles.. To carry out the latent class analysis, we used the gsem [54] function from the STATA software environment (StataCorp LLC, College Station, TX, USA), Version 17.0. To analyse differences between clusters in their sociodemographic characteristics, non-core dimensions of food-related lifestyles, and health characteristics, the chi-square test and an analysis of variance were used. Means and standard deviations (SD) are reported for continuous variables; counts and column percentages are reported for categorical variables. Discriminant analysis was used to examine the extent to which utilities for attributes levels discriminate between MFRL clusters. Estimated means and SDs are reported for all outcomes. Any two-sided *p*-value < 0.05 was considered statistically significant.

## 3. Results

### 3.1. Sample Characteristics

The structure of the analytical sample (N = 540) is comparable with the adult population of Slovenia, considering sex (51.7% male and 48.3% female), age structure (18–65 years) and geographical cohesion region (50.7% eastern region and 49.3% western region). The quota sampling matches the proportion described in the census data. Altogether, 54% had completed at least secondary school and 46% had a university degree or higher (Table 2). The average age of the participants was 42.9 years (SD 13.0) and the majority of participants (95%) declared that they were at least jointly responsible for grocery shopping in their households.

### 3.2. Consumption of Grains

To gain an insight into the respondents’ consumption of different grains, they were asked to indicate how frequently they consume eight different kinds of grains (buckwheat, barley, oats, wheat, spelt, rye, corn, and rice) on a scale from once a day to never (Supplementary Table S1). The most commonly eaten grains were wheat, corn, and rice, while buckwheat, barley, oats, and rye were typically eaten occasionally. The heterogeneity in the respondents’ health perception of certain grains was also assessed. Interestingly, grains consumed less frequently (buckwheat, spelt, barley, oats, and rye) were all perceived as healthier than more frequently consumed grains such as wheat, corn, and rice (Supplementary Figure S1).

### 3.3. Food-Related Lifestyle Segmentation

After carrying out an Exploratory Factor Analysis (EFA) to extract factors with no presumption theory (Supplementary Table S3), we conducted a Confirmatory Factor Analysis (CFA) with a maximum likelihood method to establish the convergent and discriminant validity of the constructs. The results of the CFA from the measurement model on the core modules of the MFRL instrument are presented in Table 3. The estimates of Cronbach’s alpha ranged from 0.87 to 0.93, with the internal consistency level of each structure showing satisfactory levels. All of the composite reliabilities of the constructs exceeded the cut-off value of 0.7. In terms of convergent validity, all of the confirmatory factor loadings were significant (*p* < 0.001) and exceeded 0.5, suggesting convergent validity [55]. Moreover, the AVE of all of the constructs exceeded the minimum standard of 0.5 [56], which indicates that a significant portion of that variance was explained by the constructs. Discriminant validity was also confirmed, with all AVEs greater than the squared correlations between the constructs, and the Heterotrait-Monotrait ratio of correlations lower than the cut-off value of 0.9.

A latent class cluster analysis was carried out on the 15 items of the three core components (involvement, innovation, and responsibility) of the FRL instrument, with sex and age as covariates. To determine the best underlying model, goodness-of-fit measures, such as log-likelihood, the Akaike information criterion (AIC) [57], and the Bayesian information criterion (BIC) [58] were considered. Because the number of latent variables was unknown, the analysis was repeated for a number of classes, starting with 1, until the best value for the BIC was achieved. The four-cluster model showed the lowest AIC and BIC value, whereas the LL value for the five-cluster model was the lowest (Table 4). The size, sociodemographic characteristics, and mean scores for the MFRL dimensions and indicators for the segments are reported in Table 5 and Supplementary Tables S3 and S4.

*Cluster 1* (21.7% of the sample) consisted of participants who had low scores for all three core aspects of FRL (involvement, innovation, responsibility), so this cluster is addressed as *Uninvolved*, in accordance with the classification by Grunert [59]. There were more men in this cluster compared to the whole sample, confirmed using the binominal probability test (*p* = 0.013). The participants in this cluster mostly belong in the population cohort old between 45 and65 years of age, with an average age of 48.3 years, with education at lower level, lower self-reported income, and the highest mean BMI of all four clusters. Interestingly, despite having the highest mean BMI, self-reported health status was at least good for 63%. This segment attaches low importance to healthy eating, origin, and product information.

*Cluster 2* (12.0% of the sample) had participants with the highest mean score for involvement. This segment had low engagement in innovation and use of technology for shopping scores. Based on this, they can be categorised as *Conservative* consumers [59,60]. The segment had a mean age of 40.4 (13.4), more women, and more members with a higher income and education. An important part of this cluster were students (13.8%), unemployed participants (15.4%), and households with small children (preschoolers aged up to 5 years) (15.4%).

*Cluster 3* is the smallest cluster (4.3% of the sample). This cluster is low in involvement, while high in innovation and responsibility, and it presents the highest appreciation of food origin and interest in healthy eating. As defined in previous studies [35], we refer to this cluster as *Health-conscious*. It consists of participants with the highest mean age, 55.8 (9.0) years, with more than two-thirds of participants classified in the age group 55–65 years, and no households with preschoolers. The cluster has a high proportion of women (69.6%) and includes the highest share of retired people and households with the highest self-evaluated income status and very good self-evaluated health status. 

*Cluster 4*, with 62.0% of the participants, is the largest. The cluster scored second highest in food involvement, innovation, and responsibility, and highest in both dimensions describing planning and shopping. We therefore refer to this cluster as *Moderate* consumers [59,60]. The distribution between women and men is comparable to the Slovenian national census. The age is younger, with a mean age of 40.7 (12.6) years, with more participants living in urban areas, and the highest share of participants with average (63%) and above average (17.3%) income. In comparison to the other clusters, it consists of more educated participants, a lower unemployment rate and the highest percentage of participants with self-evaluated good and very good health.

### 3.4. Results of Choice Experiment and Differences between Segments

We investigated the impact of the functional ingredients in bread, nutrition, and other claims on consumers’ bread preferences. The results of the aggregated average part-worth utilities of attribute levels and the relative attribute importance are shown in Table 6, and the means of individual part-worth utilities are presented in Supplementary Table S5. Functional ingredients had the strongest relative importance for consumers (mean relative importance of 83.9%). The relative importance of other attributes was notably lower, 3.0% for nutrition claims and 13.0% for other claims.

Considering the aggregated average part-worth utilities for functional ingredients, the consumers generally preferred bread with linseed (42.3%) or staple wheat bread without any functional ingredients (35.6%), as opposed to bread with chia seeds, Tartary buckwheat, and quinoa (−13.5%, −21.8%, and −42.6%, respectively). Surprisingly, within the nutrition claims attributes, a ‘high protein’ claim was more desirable (1.3%) than a ‘high fibre’ claim (0.6%) while, interestingly, a ‘low salt’ claim received the lowest aggregated importance (−1.8%). Among the other claims, a high aggregated average part-worth utility was observed for the ‘flour from Slovenia’ (+6.4) and ‘wholegrain’ (+1.5) claims, while lower ones were found for ‘free from additives and ‘organic,’ −5.6% and −2.2%, respectively. In relationship to the segments, we found nutrition (10.2%) and other claims (19.9%) to have the highest relative importance for the *Health-conscious* cluster, while the *Conservative* cluster presented the lowest relative importance of all for nutrition claims (2.4%). Tartary buckwheat (21.7%), low salt (4.2%), and an organic label (8.8%) were found to have the highest average aggregated importance for the *Health-conscious* cluster, with high protein (3.6%) for the *Moderate* segment. High fibre (5.4%) and wholegrain (2.5%) were among the nutrition claims which were the most important for the *Uninvolved* cluster, while among other claims, flour from Slovenia (10.2%) was the most important for the *Conservative* cluster.

The utility scores for the segments in relationship to the attributes’ levels is presented in Supplementary Table S5, showing the differences between the clusters. Observing the functional ingredients, the highest relative importance was seen for linseed, notably higher for the *Conservative* cluster (+1.66) than the others. Interestingly, all the other observed functional ingredients (chia seeds, Tartary buckwheat, and quinoa) had a lower relative importance within all the clusters, with the exception of the *Health-conscious* cluster, where Tartary buckwheat was, in addition to linseed, the most important attribute when purchasing bread (+0.38). Overall, the functional ingredient with the lowest relative importance for all the clusters was quinoa; the least interested in this attribute were the *Conservative* and *Uninvolved* clusters (−1.32 and −1.23, respectively). The ‘high fibre’ claim was the only nutritional claim which showed significant differences between segments, based on follow-up pairwise comparison tests. The highest importance for this claim was observed by the *Uninvolved* cluster (+0.12), while the other clusters attached notably less importance to it, the least by the *Uninvolved* cluster. The claim related to ‘high protein’ was the most interesting to the *Moderate* cluster (+0.08), and ‘low salt’ to the *Health-conscious* cluster (+0.07). Other claims persuaded the *Health-conscious* cluster more than the others (+0.16); the most attractive claim for this cluster was the claim ‘organic’ (+0.16), while for the *Uninvolved* cluster (−0.22) this was the least attractive claim. The claim ‘flour from Slovenia’ was the most important for the *Conservative* cluster, but it should be noted that this claim was the only one of the claims which had positive scores in all the clusters. The claim ‘wholegrain’ was interesting to the *Uninvolved* (+0.09) and *Moderate* clusters (+0.06). Interestingly, the claim ‘free from additives’ had the lowest scores from all the clusters, the least interested being the *Conservative* (−0.2) and *Health-conscious* (−0.19) clusters.

**Figure 1:**
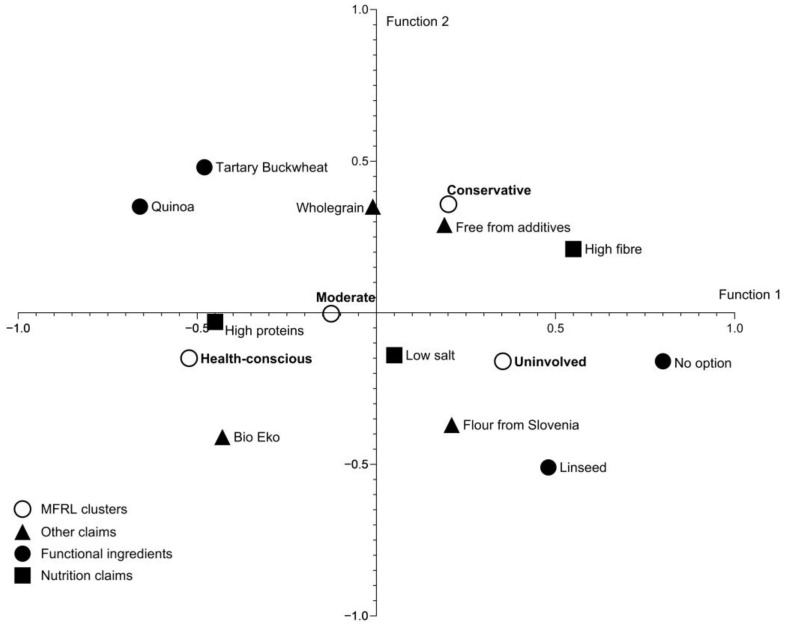
Discriminant function analysis with canonical discriminant functions classifications (modular food-related lifestyle (MFRL) segments) based on the conjoint attributes’ levels utilities.

A discriminant analysis was carried out to enable the examination of the extent to which the selected attribute levels could discriminate between the MFRL clusters. The results of the conjoint levels of individual part-worth utilities identified three canonical discriminant functions, with the first two explaining 89.1% of the variance of the between groups difference (Supplementary Table S1). The percentage of correct classification following the discriminant analysis was 62.4%. Using the test for equality of group means, we identified that several attribute levels were significantly influenced by the discrimination capacity between the FRL segments. The results show three functional ingredients (quinoa, Tartary buckwheat, and linseed) and staple wheat bread (without any functional ingredients), the nutrition claims ‘high fibre’ and ‘high protein,’ and the other claim ‘organic’ as significantly important drivers for differences between the observed segments (Supplementary Table S1). The results show that *Conservative* consumers are more likely to prefer wholegrain and free-from-additives breads compared to other segments. On the other hand, the ‘high protein’ and ‘organic’ claims are more likely to be favoured by *Health-conscious* consumers, while staple wheat bread, linseed-enriched bread, and bread produced from flour from Slovenia are more likely to be favoured by the *Uninvolved* segment (Figure 1).

## 4. Discussion

### 4.1. Food-Related Lifestyle Segments

The present study aimed to understand consumers’ preference for bread with different characteristics. When purchasing bread, consumers primarily select it based on its type, but further functional ingredients or other characteristics also come into focus [24,35,61]. We investigated the importance of functional ingredients, nutrition, and other claims in bread, using a recently developed tool for segmenting consumers based on their food-related lifestyles (FRL) [36]. The instrument measures consumers’ food-related lifestyles using three core modules: food involvement, food innovation, and food responsibility, and additional add-on modules related to planning and shopping product quality, cooking and meal preparation, consumption situations, and the motives behind the behaviour. A key idea of the new tool is to combine a core instrument with several add-on modules which can be selected according to purpose, such that segmentation solutions can be tailored to the specific needs of every application [59]. In our research, the *Planning and Shopping* and *Product Quality Aspects* modules were chosen to distinguish groups of consumers based on the role that food plays in their lives. The instrument has been shown to be related to a variety of food-related behaviours; the dimensions of the FRL or a subset of them can be directly used as predictors of food-related behaviour.

Using this method, our study resulted in the segmentation of consumers into four different clusters: *Uninvolved*, *Conservative*, *Health-conscious*, and *Moderate*. The characteristics of our clusters can be compared to those established in previous studies [35,36,59,60]: an *Uninvolved* segment of consumers, where food does not seem to have much meaning in their lives beyond ensuring survival, with low scores on the core dimensions of the FRL and add-on modules (Supplementary Tables S3 and S4); a *Conservative* segment, where food has a high preference in their lives, mostly by sticking to traditional products and ways of preparing meals, while their innovation and interest in the use of technologies for shopping, product information and food quality is quite low; a *Health-conscious* segment, with an especially high interest in food innovation and responsibility, tightly connected to product information, origin, and healthiness; and a *Moderate* cluster, characterised by high involvement and demand for quality, and by being motivated to look for product information and use technology.

### 4.2. Preferences for Functional Ingredients

Consumers consider healthiness as an important food quality aspect. As a consequence, foods containing bioactive or functional ingredients may be expected to be highly appreciated by consumers [43]. Our study confirmed the high importance of functional ingredients in comparison to nutrition and other claims when purchasing bread. Bread is one of the most important staple foods in many countries; consumption of bread with functional ingredients could therefore enhance human health performance and the prevention of disease [62]. Consumers’ interest in functional foods depends on whether they are aware of the beneficial effects; when they are, just providing information about the ingredient is enough to enable consumers to make the inference about healthiness. Linseed is a well-known functional ingredient with a longstanding tradition of use in bread [24,62], which reached the highest rate of interest in our study, while interest in the other functional ingredients (quinoa, Tartary buckwheat, and chia seeds) was lower. Consumers were obviously unfamiliar with these ingredients in bread, with the exception of *Health-conscious* consumers, who expressed a high rate of interest in Tartary buckwheat. The results of other studies have shown that the *Health-conscious* segment is more likely to prefer bread with functional ingredients [35]. It should be noted that Tartary buckwheat has characteristic sensory properties; due to the content of quercetin, it has a specific, bitter taste, which presents a barrier to consumer acceptability. Recently, several food products have been developed with various methods for masking the bitterness, and the role of Tartary buckwheat as a nutritious food ingredient has been promoted in Slovenia [21,63]. Buckwheat can provide benefits due to its content of dietary fibre, protein, minerals, and bioactive phenolic substances [64,65].

### 4.3. Preferences for Nutritional Claims

Developing and commercialising protein-enriched foods has been commonly practised by the food industry within the last decade, and has obviously impacted consumers’ perception of protein-enriched foods. The nutritional claim ‘high protein’ was the highest consumer preference of the nutritional claims in our study, especially for the *Moderate* and *Health-conscious* segments. Today, consumers are looking to increase their intake of protein for general health-and-wellness purposes. A wide range of breads are available, and consumers are obviously receptive to innovative proposals for protein-enriched bread. However, for the *Health-conscious* segment, with a mean age of 55.8 years, adequate protein intake obviously presents a particularly important food quality parameter. Recently, much communication has been in progress to make older people aware that loss of appetite is common for them, and an adequate protein intake is recommended to support the maintenance of muscle mass and strength during ageing [66,67]. 

Wholegrain products are recommended as a preferred way for securing a sufficient intake of dietary fibre in several dietary recommendations [68]. In our study, the group of *Uninvolved* consumers were notably the most willing to purchase bread with a high fibre claim or wholegrain bread, despite their low interest in healthy eating. Obviously, the claims ‘wholegrain’ and ‘high in fibre’ are commonly known and consumers are familiar with them. As reported by Foster et al., many consumers nowadays are well-informed about the importance of whole grains in their diets [69]. 

The nutritional claim ‘low in salt,’ surprisingly, did not get much attention. The *Health-conscious* consumers expressed the highest interest in purchasing bread low in salt. In this segment, women prevailed and, according to literature studies, women have more favourable attitudes towards health [70]. The participants of *Moderate* cluster were not interested in bread low in salt. The mean age for this cluster was low (only 40.7 years) and their self-reported health status was the highest in comparison to all the other clusters. It could be assumed that when purchasing bread, taste plays a more important role than healthiness for this cluster. It has already been discussed that the potential of taste to influence consumer food preferences can be stronger than the potential of nutrients with a positive impact on health [2,33,71]. However, despite the fact that lowering salt intake is a key priority of many reformulation strategies and public health interventions [72], it is necessary to further communicate this topic to consumers, to increase their awareness of the health benefits of lowering salt intake and to strengthen their willingness to eat bread with lower salt content. It should be also noted that modest reductions in salt content in bread can be achieved without notably affecting the sensory properties of the bread [25], while even greater improvements are possible with gradual salt content reduction over a longer time period [18].

### 4.4. Preferences for Other Claims

Other claims on foods include statements referring to health, processing, quality and additives [73], for example, statements about a food product’s characteristics, production methods and marketing trends (i.e., natural, free from additives, free from preservatives, organic, GMO-free, no artificial colours, etc.). In this study we tested four such claims often found on bread sold on the Slovenian market: ‘organic,’ ‘flour from Slovenia,’ ‘wholegrain’ and ‘free from additives.’ Concerning the claim ‘flour from Slovenia,’ our results indicate that consumers from all clusters had a positive attitude towards bread prepared with Slovenian flour. Interestingly, the *Uninvolved* and *Conservative* clusters expressed the highest interest in this attribute, despite their low interest in healthy eating and origin, but obviously when bread as a staple food is in focus, the origin of flour becomes important. Besides, studies have also shown that no matter the level of involvement with the purchase, domestic products will more often convey feelings of reward to consumers, and will be preferred [32]. However, consumers today also pay attention to sustainability attributes, such as local and organic [74]. In addition, consumers associate organic production not only with concern for the environment but also with health and good taste [75,76,77]. The organic claim on bread products was not positively valued by all of the clusters, but only by the *Conservative* and *Health Conscious* consumers, who also expressed the highest interest in organic bread. This is in line with the high FRL responsibility score for this cluster. Contrary to expectations, the claim ‘free from additives’ had the lowest valuation from all of the clusters in our study. Findings from a study by Rybak et al. [10] suggest that processing claims also lead to inferences on nutrition, a clean label, and healthfulness, while nutritional claims only influence nutrition evaluations. In fact, in our study as well, processing claim manipulations on bread appear stronger than those emanating from nutritional claims.

### 4.5. Study Limitations

Some study limitations need to be mentioned. First, the analysis only focused on bread, and the study results should therefore not be directly applied to other food matrices. The second concern is related to the fact that our study was based on a hypothetical choice experiment, whereas studying choices in a real-life environment (in which the choice of bread actually takes place, with the use of real products) would lead to results with higher validity. We should also mention that in giving the study design an objective, we did not focus on some bread properties that are well recognised as important to consumers, including product price. In addition, the number of levels in the choice-based conjoint experiment differed between attributes, and that this could influence the importance assigned to the attributes [78]. Our study was conducted in Slovenia, where bread consumption is very common; the study findings should be used with caution for populations with different cultural backgrounds. We should also note that although study sample size was comparable with similar studies, such sample size has limited power to provide meaningful insights into specific smaller subsamples, which would be interesting to further explore. This is also limiting our ability to generalize study findings for specific population groups. Future studies are needed to verify reported results and investigate the bread preferences of different generations, as our analysis shows that different age cohorts present different food-related lifestyles which further influence different bread attributes perceptions.

## 5. Conclusions

The study results demonstrate a strong consumer awareness of the benefits of the improved composition of bread and reaffirm interest in support of alternative and traditional grains with functional characteristics in bread production. Functional ingredients improved nutritional composition, and other product-related characteristics were recognised as added value for consumers. The study also validated the new modular FRL instrument on the sample of the Slovenian population, and highlighted four consumer segments, which were useful in explaining the variability in consumers’ preferences in bread attributes. The study results support the incorporation of alternative grains in bread production, and offer insights into consumer preferences, which could support the marketing of improved or new bread products in the competitive market. The study also provides valuable information for undertaking further activities referring to public health priorities. We observed a lack of consumer interest related to messages of high public health concern, such as salt content in bread, which urges more efficient educational interventions for the general population. Further research should be focused also into specific population groups, to enable development of tailored interventions.

## Figures and Tables

**Table 1:** Attributes of bread and their levels used in the conjoint analysis.

Attribute	Attribute Levels
Functional ingredient	No functional ingredient
	Chia seeds
	Linseed
	Quinoa
	Tartary buckwheat
Nutrition claim	Low salt
	High fibre
	High protein
Other claim	Organic (bio/eco)
	Free from additives
	Flour from Slovenia
	Wholegrain

**Table 2:** Socio-demographic and health related characteristics of the sample (N = 540).

Variable	Level	N (%)
Sex	Male	279 (51.7)
	Female	261 (48.3)
Age: Mean (SD)		42.9 (13.0)
Age classes *	18–29	105 (19.4)
	30–44	170 (31.5)
	45–54	129 (23.9)
	55–65	136 (25.2)
Geographical cohesion region	East	274 (50.7)
	West	266 (49.3)
Household location	Urban	298 (55.2)
	Rural	242 (44.8)
Education	Lower level	291 (53.9)
	Higher level	249 (46.1)
Self-evaluated financial status	Below average	120 (22.2)
	Average	329 (60.9)
	Above average	91 (16.9)
Employment	Employed	371 (69.0)
	Retired	56 (10.4)
	Housekeeping member	8 (1.5)
	Student	49 (9.1)
	Unemployed	54 (10.0)
Household structure	Household with preschoolers	72 (13.3)
	Household with members aged 5–65	406 (75.2)
	Household with at least one person older than 65	62 (11.5)
BMI: Mean (SD)		26.1 (6.8)
Self-evaluated health status	Poor and very poor	14 (2.6)
	Average	150 (27.8)
	Good and very good	376 (69.6)

Notes: BMI—body mass index calculated from self-reported height and weight of attendants; SD—standard deviation; (*) Developed age classes have only an informative (analytical) nature, they are not part of the sampling frame.

**Table 3:** Results of constructs’ convergent and discriminant validity using confirmatory factor analysis on the core modules of the food-related lifestyle scale (N = 540).

MFRL Core Dimensions and Items	Standardised Factor Loadings	Average Inter-Item Correlation	CR	AVE
Involvement (Cronbach’s alpha = 0.87)		0.59	0.88	0.60
*Food and drink are an important part of my life*	0.86			
*Eating and drinking are a continuous source of joy for me*	0.77			
*Eating and food is an important part of my social life*	0.78			
*I just love good food*	0.80			
*Decisions on what to eat and drink are very important for me*	0.66			
Innovation (Cronbach’s alpha = 0.93)		0.73	0.93	0.73
*I love to try recipes from different countries*	0.87			
*Recipes and articles on food from other culinary traditions encourage me to experiment in the kitchen*	0.86			
*I look for ways to prepare unusual meals*	0.86			
*I like to try out new recipes*	0.88			
*I like to try new foods that I have never tasted before*	0.82			
Responsibility (Cronbach’s alpha = 0.92)		0.69	0.92	0.69
*I try to choose food produced with a minimal impact on the environment*	0.89			
*I try to choose food that is produced in a sustainable way*	0.82			
*I am concerned about the conditions under which the food I buy is produced*	0.81			
*It is important to understand the environmental impact of our eating habits*	0.81			
*I try to buy organically produced foods if possible*	0.81			
**Estimated Correlations**	**SC**		**HTMT**	
Involvement vs. Innovation	0.225		0.5	
Involvement vs. Responsibility	0.140		0.5	
Innovation vs. Responsibility	0.280		0.48	

Notes: MFRL—a modular food-related lifestyle instrument; AVE—Average Variance Extracted; CR—Composite Reliability; SC—Squared correlation; HTMT—Heterotrait-Monotrait ration of correlations.

**Table 4:** Model selection for latent class segmentation based on the core modules of the MFRL.

No. of Latent Classes	Log-Likelihood	df	AIC	BIC
1	−2183.5	6	4378.9	4404.7
2	−2158.8	12	4341.5	4393.0
3	−2142.9	18	4321.9	4399.1
4	−2131.5	24	4311.0	4414.0
5	−2127.6	30	4315.3	4444.0

Notes: MFRL—a modular food related lifestyle instrument; BIC—Bayesian information criteria; AIC—Akaike information criterion.

**Table 5:** Description of MFRL clusters based on sociodemographic, individuals’ health-related attributes, and MFRL N (%).

Variables	Uninvolved	Conservative	Health-Conscious	Moderate
Size	117 (21.7)	65 (12.0)	23 (4.3)	335 (62.0)
Sex				
Male	71 (60.7)	26 (40.0)	7 (30.4)	175 (52.2)
Female	46 (39.3)	39 (60.0)	16 (69.6)	160 (47.8)
Age: Mean (SD)	48.3 (11.6)	40.4 (13.4)	55.8 (9.0)	40.7 (12.6)
Age classes				
18–29	12 (10.3)	17 (26.2)	1 (4.3)	75 (22.4)
30–44	28 (23.9)	24 (36.9)	2 (8.7)	116 (34.6)
45–54	36 (30.8)	10 (15.4)	3 (13.0)	80 (23.9)
55–65	41 (35.0)	14 (21.5)	17 (73.9)	64 (19.1)
Household location				
Urban	63 (53.8)	35 (53.8)	12 (52.2)	188 (56.1)
Rural	54 (46.2)	30 (46.2)	11 (47.8)	147 (43.9)
Education *				
Lower level	72 (61.5)	36 (55.4)	14 (60.9)	169 (50.4)
Higher level	45 (38.5)	29 (44.6)	9 (39.1)	166 (49.6)
Self-evaluated financial status				
Below average	32 (27.4)	14 (21.5)	8 (34.8)	66 (19.7)
Average	67 (57.3)	41 (63.1)	10 (43.5)	211 (63.0)
Above average	18 (15.4)	10 (15.4)	5 (21.7)	58 (17.3)
Employment				
Employed	76 (65.0)	38 (58.5)	13 (56.5)	244 (73.3)
Retired	21 (17.9)	7 (10.8)	6 (26.1)	22 (6.6)
Housekeeping member	2 (1.7)	1 (1.5)	1 (4.3)	4 (1.2)
Student	5 (4.3)	9 (13.8)	/	35 (10.5)
Unemployed	13 (11.1)	10 (15.4)	3 (13.0)	28 (8.4)
Household structure				
With preschooler	12 (10.3)	10 (15.4)	/	50 (14.9)
With members between aged 5–65	93 (79.5)	47 (72.3)	18 (78.3)	248 (74.0)
With at least one older than 65	12 (10.3)	8 (12.3)	5 (21.7)	37 (11.0)
BMI: Mean (SD)	27.6 (11.3)	25.2 (4.6)	24.1 (6.3)	25.9 (4.8)
Self-evaluated health status				
Poor and very poor	5 (4.3)	2 (3.10)	3 (13.0)	4 (1.2)
Average	38 (32.5)	20 (30.8)	4 (17.4)	88 (26.3)
Good and very good	74 (63.2)	43 (66.2)	16 (69.6)	243 (72.5)
MFRL modules				
Involvement	3.79 (0.79) ^a^	6.03 (0.67) ^b^	3.63 (0.58) ^a^	5.70 (0.83) ^c^
Innovation	2.79 (1.00) ^a^	2.58 (0.85) ^a^	5.37 (1.02) ^b^	5.29 (1.00) ^b^
Responsibility	4.17 (1.38) ^a^	4.47 (1.39) ^ab^	5.27 (1.24) ^bc^	5.15 (1.21) ^c^
Planning and Shopping: Mean (SD)				
Use of technology for shopping	2.34 (1.27) ^a^	2.30 (1.21) ^a^	2.45 (1.58) ^ab^	3.24 (1.69) ^b^
Product information	3.52 (1.58) ^a^	3.77 (1.85) ^ab^	4.62 (1.40) ^bc^	4.71 (1.46) ^c^
Product quality aspects: Mean (SD)				
Origin	4.73 (1.49) ^a^	5.01 (1.51) ^a^	5.93 (1.04) ^b^	5.56 (1.24) ^b^
Healthy eating	4.11 (1.38) ^a^	4.31 (1.51) ^a^	5.21 (1.14) ^b^	4.99 (1.28) ^b^

Note: SD—standard deviation; BMI—body mass index; MFRL—a modular food-related lifestyle instrument; * Lower level of education is completed secondary school or lower; Higher education is university degree or higher. Values in the same row and sub-table not sharing the same superscript letters are significantly different at *p* < 0.05 in the two-sided test of equality for column means. Tests assume equal variances. Tests are adjusted for all pairwise comparisons within a row of each innermost sub-table using the Bonferroni correction.

**Table 6:** The attributes’ relative importance and aggregated average part-worth utilities of all the attribute levels for the whole sample and for different clusters.

Attributes and Levels	Relative and Aggregated Average Importance (%)				
	All	Uninvolved	Conservative	Health-Conscious	Moderate
**Functional ingredient**	**83.9**	**81.4**	**81.6**	**69.9**	**81.7**
No functional ingredient	35.6	43.3	39.7	6.9	28.0
Linseed	42.3	36.3	42.7	29.4	40.8
Chia seeds	−13.5	−20.6	−15.3	−17.7	−7.8
Tartary buckwheat	−21.8	−20.8	−28.0	21.7	−20.2
Quinoa	−42.6	−38.1	−39.1	−40.4	−40.9
**Nutrition claims**	**3.0**	**6.2**	**2.4**	**10.2**	**6.4**
High fibre	0.6	3.6	0.8	−6.4	−0.7
High protein	1.3	−2.5	−1.4	2.2	3.6
Low salt	−1.8	−1.1	0.6	4.2	−2.9
**Other claims**	**13.1**	**12.4**	**16.0**	**19.9**	**11.9**
Flour from Slovenia	6.4	5.5	10.2	3.1	5.2
Wholegrain	1.5	2.8	−4.6	−1.1	2.5
Organic (bio/eco)	−2.2	−6.9	0.2	8.8	−1.0
Free from additives	−5.6	−1.4	−5.8	−10.8	−6.7

## Data Availability

All participants were fully informed why the research is being conducted and how their data will be used, and consented to study participation, knowing about the ability to withdraw from the study at any time. There was no coercion to participate. The data received from the online consumer panel operator did not contain any personal identifications. All results are presented in aggregated form. No risks were identified for study participants. Ethical approval is not required by national law. The data presented in this study are available upon request from the corresponding author.

## Supplementary Materials

The following supporting information can be downloaded at https://www.mdpi.com/article/10.3390/foods12203766/s1: Supplementary Figure S1: Heterogeneity in health perception of selected grains (N = 540); Supplementary Table S1: Grain type consumption frequency in numbers and percentages (N = 540); Supplementary Table S2: Test of equality of groups means and pooled within-groups correlations between discriminating variables and the standardized canonical discriminant functions (structure matrix); Supplementary Table S3: Factor analysis of three core MFRL modules and means (SD) per sample and LCA segments; Supplementary Table S4: Factor analysis for add-on MFRL modules and means (SD) per sample and LCA segments; Supplementary Table S5: The part-worth utilities of attribute levels and their relative importance per total and per individual cluster; Supplementary Tool S5: Survey questionnaire.
