# Supplementary material for: Consumers’ Preferences towards Bread Characteristics Based on Food-Related Lifestyles: Insights from Slovenia
Source: Foods. 2023 Oct 13;12(20):3766. doi: 10.3390/foods12203766 (PMC10606115; doi:10.3390/foods12203766)
Supplement: Supplementary file 1 [file foods-12-03766-s001.zip › foods-2553977-supplementary.pdf]

foods

MDPI

Consumers' Preferences towards Bread Characteristics Based on Food-Related Lifestyles: Insights from Slovenia

Anita Kušar [1,*], Igor Pravst [1,2,3], Urška Pivk Kupirovič [1], Klaus G. Grunert [4,5], Ivan Kreft [1] and Hristo Hristov [1]

Supplementary Table S1: Grain type consumption frequency in numbers and percentages (N = 540).

Grains	Once a day or more	More times per week	Once a week	1-3 times per month	Less than 1 time per month	Never
Buckwheat	4 (0.7)	43 (8.0)	92 (17.0)	187 (34.6)	162 (30.0)	52 (9.6)
Barley	3 (0.6)	21 (3.9)	61 (11.3)	199 (36.9)	206 (38.1)	50 (9.3)
Oats	15 (2.8)	50 (9.3)	69 (12.8)	115 (21.3)	164 (30.4)	127 (23.5)
Wheat	101 (18.7)	246 (45.6)	97 (18.0)	59 (10.9)	24 (4.4)	13 (2.4)
Spelt	10 (1.9)	50 (9.3)	84 (15.6)	135 (25.0)	174 (32.2)	87 (16.1)
Rye	4 (0.7)	31 (5.7)	62 (11.5)	112 (20.7)	198 (36.7)	133 (24.6)
Corn	10 (1.9)	65 (12.0)	140 (25.9)	188 (34.8)	115 (21.3)	22 (4.1)
Rice	12 (2.2)	89 (16.5)	242 (44.8)	150 (27.8)	37 (6.9)	10 (1.9)

Note: The values in paranthesis are percentages.

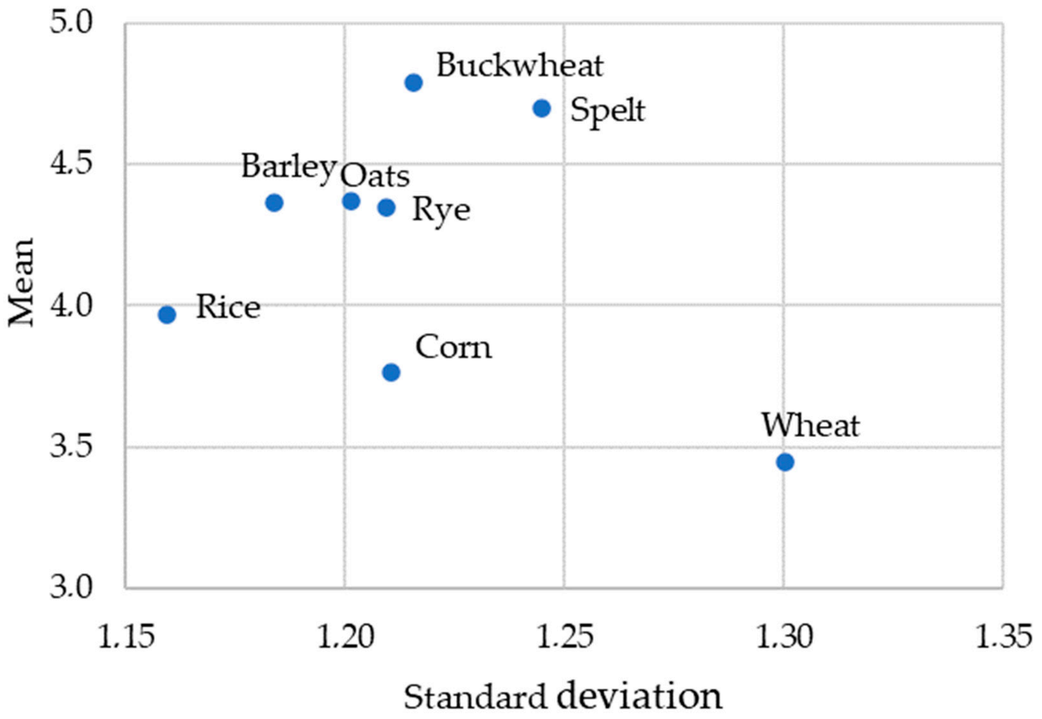

Supplementary Figure S1. Heterogeneity in health perception of selected grains (N= 540).

Supplementary Table S2. Test of equality of groups means and pooled within-groups correlations between discriminating variables and the standardized canonical discriminant functions (structure matrix).

Variables	Wilks' lambda	F	p value	Structure matrix		
				Function 1 (63.2% of variance explained)	Function 2 (25.9% of variance explained)	Function 3 (10.9% of variance explained)
No SF ingredient	0.966	6.254	<0.001	0.80*	-0.16	0.16
Quinoa	0.974	4.7	0.003	-0.66*	0.35	-0.14
High fibre	0.983	3.063	0.028	0.55*	0.21	-0.05
Organic (bio/eco)	0.986	2.558	0.05	-0.43*	-0.41	0.25
Linseed	0.982	3.296	0.02	0.48	-0.51*	-0.08
Flour from Slovenia	0.994	1.013	N.S.	0.21	-0.37*	0.16
Wholegrain	0.997	0.599	N.S.	-0.01	0.35*	-0.27
Free from additives	0.996	0.659	N.S.	0.19	0.29*	-0.02
Low in salt	0.995	0.849	N.S.	0.05	-0.14	0.67*
High protein	0.987	2.433	0.064	-0.45	-0.03	-0.56*
Tartary Buckwheat	0.981	3.53	0.015	-0.48	0.48	0.50*

Notes: N.S. – not significant. * Largest absolute correlation between each variable and any discriminant function. The coordinates of MFRL segments in three function distribution plots are: Uninvolved (0.353; -0.160; -0.032), Conservative (0.201; 0.358; -0.081), Health-conscious (-0.523; -0.150; -0.389), Moderate (-0.126; -0.003; 0.054).

Supplementary Table S3: Factor analysis of three core MFRL modules and means (SD) per sample and LCA segments.

MFRL core modules and measuring items	Factor Loading	Mean (SD)	Uninvolved Mean (SD)	Conservative Mean (SD)	Health-conscious Mean (SD)	Moderate Mean (SD)
**Involvement (Variance explained = 20.2%)**		5.2 (1.2)				
Food and drink are an important part of my life	0.85	5.4 (1.4)	3.9 (1.0)	6.4 (0.8)	3.5 (1.0)	5.9 (1.0)
Eating and drinking are a continuous source of joy for me	0.76	4.7 (1.6)	3.2 (1.1)	5.7 (1.3)	2.3 (1.3)	5.2 (1.3)
Eating and food is an important part of my social life	0.74	4.9 (1.5)	3.5 (1.1)	5.6 (1.3)	2.8 (1.4)	5.4 (1.2)
I just love good food	0.75	5.7 (1.3)	4.3 (1.3)	6.5 (0.7)	4.6 (1.6)	6.2 (0.9)
Decisions on what to eat and drink are very important for me	0.56	5.4 (1.4)	4.1 (1.4)	5.9 (1.1)	5.0 (1.7)	5.8 (1.1)
**Innovation (Variance explained = 23.9%)**		4.4 (1.6)				
I love to try recipes from different countries	0.83	4.3 (1.8)	2.7 (1.1)	2.3 (1.1)	5.2 (1.5)	5.2 (1.3)
Recipes and articles on food from other culinary traditions encourage me to experiment in the kitchen	0.80	4.2 (1.8)	2.7 (1.2)	2.3 (1.2)	5.1 (1.5)	5.1 (1.4)
I look for ways to prepare unusual meals	0.81	4.1 (1.8)	2.6 (1.2)	2.1 (1.0)	5.1 (1.5)	5.0 (1.4)
I like to try out new recipes	0.79	4.8 (1.7)	3.1 (1.2)	3.2 (1.6)	5.9 (1.1)	5.7 (1.1)
I like to try new foods that I have never tasted before	0.75	4.6 (1.8)	3.0 (1.4)	2.9 (1.5)	5.5 (1.3)	5.5 (1.2)
**Responsibility (Variance explained = 24.0%)**		4.9 (1.3)				
I try to choose food produced with minimal impact on the environment	0.86	4.6 (1.6)	4.0 (1.5)	4.0 (1.8)	5.1 (1.5)	5.0 (1.5)
I try to choose food that is produced in a sustainable way	0.78	4.6 (1.5)	4.0 (1.6)	4.4 (1.5)	5.0 (1.5)	4.9 (1.4)
I am concerned about the conditions under which the food I buy is produced	0.77	5.2 (1.5)	4.4 (1.5)	5.0 (1.7)	5.6 (1.5)	5.5 (1.3)
It is important to understand the environmental impact of our eating habits	0.77	5.1 (1.5)	4.4 (1.5)	4.7 (1.5)	5.1 (1.8)	5.4 (1.3)
I try to buy organically produced foods if possible	0.79	4.7 (1.7)	4.1 (1.6)	4.3 (1.8)	5.5 (1.5)	5.0 (1.6)

Notes: All three core MFRL dimensions together explain 68% of the variance of the scale.

Supplementary Table S4: Factor analysis for add-on MFRL modules and means (SD) per sample and LCA segments.

Dimensions and corresponding measures	Mean (SD)	Factor 1 loadings	Factor 2 loadings	Uninvolved Mean (SD)	Conservative Mean (SD)	Health-conscious Mean (SD)	Moderate Mean (SD)
**Planning and Shopping: (Variance explained by two factors = 100%)**							
Use of technology for shopping (Cronbach's alpha = 0.85)	2.9 (1.6)						
Shopping apps assist me with food purchasing choices	2.9 (1.9)	0.787		2.4 (1.5)	2.5 (1.8)	2.5 (2.0)	3.2 (1.9)
I use recipe apps to generate shopping lists	2.7 (1.8)	0.807		2.2 (1.3)	1.8 (1.1)	2.7 (1.8)	3.0 (1.9)
I use my smartphone for information when shopping	3.1 (1.9)	0.808		2.5 (1.6)	2.6 (1.7)	2.2 (1.8)	3.5 (1.9)
Product information (Cronbach's alpha = 0.89)	4.3 (1.6)						
I compare product information on food labels to decide which brand to buy	4.2 (1.8)		0.78	3.5 (1.6)	3.5 (1.9)	4.6 (1.5)	4.6 (1.7)
I read the nutritional panel and ingredients list to select the most nutritious food	4.0 (1.9)		0.786	3.2 (1.7)	3.5 (2.2)	3.9 (1.8)	4.4 (1.8)
To me product information is of high importance. I need to know what the product contains	4.8 (1.7)		0.686	3.9 (1.8)	4.4 (2.0)	5.3 (1.6)	5.1 (1.5)
**Product quality aspects: (Variance explained by two factors = 98.6%)**							
Origin (Cronbach's alpha = 0.88)	5.3 (1.4)						
It is important for me to know where the food I buy comes from	5.1 (1.6)	0.667		4.5 (1.6)	4.8 (1.9)	5.5 (1.5)	5.4 (1.4)
I prefer to eat food which is locally produced	5.4 (1.5)	0.815		4.8 (1.5)	5.1 (1.7)	6.0 (1.2)	5.6 (1.4)
I prefer to purchase food grown or produced in my home country	5.5 (1.5)	0.836		4.9 (1.6)	5.2 (1.7)	6.3 (0.9)	5.7 (1.4)
Healthy eating (Cronbach's alpha = 0.82)	4.7 (1.4)						
I try to avoid food products with artificial additives or preservatives	5.1 (1.7)		0.717	4.4 (1.7)	4.6 (1.9)	6.0 (1.2)	5.3 (1.6)
I specifically buy some foods for their health benefits rather than taste	4.4 (1.8)		0.585	3.9 (1.7)	4.0 (2.1)	4.6 (1.9)	4.5 (1.8)
I generally believe in the benefits of functional foods	4.6 (1.5)		0.551	3.8 (1.4)	4.2 (1.7)	5.3 (1.3)	4.8 (1.4)
I look for products which are lower in sugar and/or salt	5.0 (1.8)		0.679	4.3 (1.8)	4.5 (2.0)	4.9 (2.0)	5.3 (1.7)

Supplementary Table S5: The part-worth utilities of attribute levels and their relative importance per total and per individual cluster.

Attribute	Level	All<br>Mean (SD)	Uninvolved<br>Mean (SD)	Conservative<br>Mean (SD)	Health-conscious<br>Mean (SD)	Moderate<br>Mean (SD)
**Pseudocereals**	**Relative importance (%)**	**83.91**	**81.42**	**81.66**	**69.89**	**81.70**
	Without pseudocereal present	0.88 (2.02)	1.4 (1.97)[a]	1.34 (1.98)[ab]	0.12 (2.04)[b]	0.66 (2.00)[bc]
	Linseed	1.04 (1.46)	1.17 (1.47)	1.44 (1.66)	0.52 (1.48)	0.96 (1.40)
	Chia seeds	-0.33 (1.6)	-0.66 (1.56)[a]	-0.52 (1.52)[ab]	-0.31 (1.63)[ab]	-0.18 (1.61)[b]
	Tartary buckwheat	-0.54 (1.8)	-0.67 (1.79)[ab]	-0.95 (1.68)[a]	0.38 (2.18)[b]	-0.47 (1.78)[ab]
	Quinoa	-1.05 (0.99)	-1.23 (1.00)[ab]	-1.32 (0.84)[a]	-0.71 (1.05)[ab]	-0.96 (1.00)[b]
**Nutritional claims**	**Relative importance (%)**	**3.04**	**6.19**	**2.37**	**10.23**	**6.38**
	High fibre	0.01 (0.46)	0.12 (0.48)[a]	0.03 (0.41)[ab]	-0.11 (0.49)[ab]	-0.02 (0.46)[b]
	High protein	0.03 (0.63)	-0.08 (0.60)	-0.05 (0.52)	0.04 (0.44)	0.08 (0.66)
	Low salt	-0.04 (0.55)	-0.04 (0.56)	0.02 (0.44)	0.07 (0.59)	-0.07 (0.56)
**Processing claims**	**Relative importance (%)**	**13.04**	**12.38**	**15.98**	**19.89**	**11.91**
	Flour from Slovenia	0.16 (0.98)	0.18 (0.93)	0.34 (1.03)	0.05 (0.83)	0.12 (1.00)
	Wholegrain	0.04 (1.28)	0.09 (1.27)	-0.15 (1.20)	-0.02 (1.16)	0.06 (1.31)
	Organic (bio/eco)	-0.06 (0.81)	-0.22 (0.72)	0.01 (0.88)	0.16 (0.90)	-0.02 (0.81)
	Free from additives	-0.14 (0.85)	-0.04 (0.83)	-0.2 (0.86)	-0.19 (0.91)	-0.16 (0.85)

Notes: Mean relative importance for each attribute. Values in the same row and sub-table not sharing the same subscript letters are significantly different at $p< 0.05$ in the two-sided test of equality for column means. Tests assume equal variances. Tests are adjusted for all pairwise comparisons within a row of each innermost sub-table using the Bonferroni correction.
